# Genomic insights into the lifestyles, functional capacities and oleagenicity of members of the fungal family *Trichosporonaceae*

**DOI:** 10.1038/s41598-020-59672-2

**Published:** 2020-02-17

**Authors:** Habibu Aliyu, Olga Gorte, Pieter de Maayer, Anke Neumann, Katrin Ochsenreither

**Affiliations:** 1 Institute of Process engineering in Life Science 2: Technical Biology, Karlsruhe Institute of Technology, Karlsruhe, Germany; 2 School of Molecular & Cell Biology, Faculty of Science, University of the Witwatersrand, WITS 2050 Johannesburg, South Africa

**Keywords:** Molecular evolution, Taxonomy, Genomics

## Abstract

*Trichosporonaceae* incorporates six genera of physiologically and ecologically diverse fungi including both human pathogenic taxa as well as yeasts of biotechnological interest, especially those oleagenic taxa that accumulate large amounts of single cell oils (SCOs). Here, we have undertaken comparative genomic analysis of thirty-three members of the family with a view to gain insight into the molecular determinants underlying their lifestyles and niche specializations. Phylogenomic analysis revealed potential misidentification of three strains which could impact subsequent analyses. Evaluation of the predicted proteins coding sequences showed that the free-living members of the family harbour greater numbers of carbohydrate active enzymes (CAZYmes), metallo- and serine peptidases compared to their host-associated counterparts. Phylogenies of selected lipid biosynthetic enzymes encoded in the genomes of the studied strains revealed disparate evolutionary histories for some proteins inconsistent with the core genome phylogeny. However, the documented oleagenic members distinctly cluster based on the constitution of the upstream regulatory regions of genes encoding acetyl-CoA carboxylase (ACC), ATP-citrate synthase (ACS) and isocitrate dehydrogenase [NADP] (ICDH), which are among the major proteins in the lipid biosynthetic pathway of these yeasts, suggesting a possible pattern in the regulation of these genes.

## Introduction

The basidiomycetous fungal family *Trichosporonaceae* belongs to the order Trichosporonales, the class Tremellomycetes, and subphylum Agaricomycotina and incorporates morphologically and physiologically diverse, aromatic compound-assimilating yeasts^1^. Recently the taxonomy of this family was revised to include six genera, namely *Apiotrichum, Cutaneotrichosporon, Effuseotrichosporon, Haglerozyma, Trichosporon* (type genus) and *Vanrija*. This revision was based on phylogenetic analysis of seven markers, namely LSU (D1/D2 domains) and SSU rRNA, the Internal Transcribed Spacer (ITS) and the protein coding genes RPB1, RPB2, TEF1 and CYTB and a combination of morphological, biochemical and physiological characteristics^1,2^. Members of the *Trichosporonaceae* show a global distribution and have been recovered from a wide range of environments. *Cutaneotrichosporon* spp. are most frequently associated with a human host, and may represent opportunistic human pathogens. *Trichosporon* spp. form part of the natural microflora on human and animal skin and result in a non-serious mycosis of hair termed white piedra^3^. However, they have also been implicated in trichosporonosis, a collection of opportunistic infections caused by a number of species, including *Trichosporon asahii*, *T. asteroides* and *T. ovoides*^4^. By contrast *Apiotrichum* and *Vanrija* spp. are generally free-living and have been isolated from water bodies, food sources and rotten wood (Table 1).

**Table 1:** Genome features of thirty-three *Trichosporonaceae* and two outgroup species included in the analysis.

Strains	Accession	BioProject	Isolation source/locality	Sequencing technology	Assembly Size (bp)	# Scaffolds	N50 (bp)	% G + C	# Genes	# tRNA	# Proteins	Unique Proteins	Prots atleast 1 orthologue	Comp*	Dup^¥^
*Apiotrichum brassicae* JCM 1599^T^	BCJI00000000	PRJDB3695	Cabbage purchased in a market/Tokyo, Japan	HiSeq. 2500	23,647,732	16	2,410,059	56.47	8,334	422	7,912	1,204	6,708	91.3	0.2
*Apiotrichum domesticum* JCM 9580^T^	BCFW00000000	PRJDB3573	Rotten wooden sideboard (pneumonitis patient), Japan	HiSeq. 2500	24,510,922	28	3,306,260	58.52	8,536	379	8,157	582	7,575	91.1	0.6
*Apiotrichum gamsii* JCM 9941^T^	BCJN00000000	PRJDB3703	Moist humus around roots/Colombia	HiSeq. 2500	24,609,388	29	3,284,570	61.14	8,608	385	8,223	1,628	6,595	96.3	1.2
*Apiotrichum gracile* JCM 10018	BCJO00000000	PRJDB3704	Sour milk/Germany	HiSeq. 2500	24,114,851	17	4,554,691	59.23	9,556	918	8,638	1,677	6,961	90.2	0.5
*Apiotrichum laibachii* JCM 2947^T^	BCKV00000000	PRJDB3730	Soil	HiSeq. 2500	30,616,633	26	2,819,049	59.63	11,708	1,530	10,178	1,886	8,292	92.8	0.5
*Apiotrichum montevideense* JCM 9937	BCFV00000000	PRJDB3572	Water purification tank/Montevideo, Uruguay	HiSeq. 2500	24,872,216	61	1,972,470	58.17	8,550	375	8,175	599	7,576	91.9	0.4
*Apiotrichum porosum* DSM 27194	RSCE00000000	PRJNA506377	Grassland /Germany	PacBio	25,479,456	32	1,376,709	59.15	9,729	576	9,153	555	8,598	96.7	0.9
*Apiotrichum porosum* JCM 1458T	BCJG00000000	PRJDB3693	Exudate of Taxus baccata/Hamburg, Germany	HiSeq. 2500	25,989,348	37	3,276,928	59.05	9,816	546	9,270	662	8,608	97.2	0.9
*Apiotrichum veenhuisii* JCM 10691T	BCKJ00000000	PRJDB3717	Buffalo dung/Minturno, Campania, Italy	HiSeq. 2500	31,617,680	35	2,551,142	59.59	10,127	1,019	9,108	1,778	7,330	92.2	0.7
*Cutaneotrichosporon cutaneum* ACCC 20271	LTAL00000000	PRJNA313001	Hangzhou oil refinery/China	Illumina MiSeq	30,717,177	21	5,629,136	57.13	9,586	878	8,708	1,386	7,322	94.1	0.4
*Trichosporon akiyoshidainum* HP2023	PQXP00000000	PRJNA428315	Ryzosphere/Las Yungas rainforest, Argentina	Illumina MiSeq	30,042,643	1067	53,521	60.69	11,492	1,292	10,200	1,746	8,454	90.5	4.3
*Cutaneotrichosporon arboriformis* JCM 14201^T^	BEDW00000000	PRJDB5900	Urine of chronic renal failure patient/Hokkaido, Japan	Illumina MiSeq	19,894,493	28	1,599,305	60.59	7,697	143	7,554	1,357	6,197	88.7	0.5
*Cutaneotrichosporon curvatus* JCM 1532^T^	BCJH00000000	PRJDB3694	Sputum of tubercular patient/The Netherlands	HiSeq. 2500	18,637,344	75	571,590	57.89	7,137	151	6,986	293	6,693	88.4	0.3
*Cutaneotrichosporon curvatus* SBUG-Y 855	LDEP00000000	PRJNA281029	Sputum of tubercular patient/Netherlands	Illumina GAIIx	16,443,618	354	62,631	59.41	6,628	151	6,477	246	6,231	80.8	0.6
*Cutaneotrichosporon cutaneum* B3	LRUG00000000	PRJNA310294	Soil/Nanchang, Jiangxi, China	Illumina HiSeq	38,696,417	592	116,762	60.30	14,851	448	14,403	1,004	13,399	94.1	**55.7**
*Cutaneotrichosporon cutaneum* JCM 1462	BCKU00000000	PRJDB3729	Probably human clinical specimen	HiSeq. 2500	23,155,501	98	744,446	61.97	9,635	399	9,236	1,756	7,480	88.3	0.4
*Cutaneotrichosporon cyanovorans* JCM 31833	BEDZ00000000	PRJDB5903	Cyanide contaminated soil /Sasolburg, South Africa	Illumina MiSeq	19,941,766	90	582,111	58.01	7,086	150	6,936	993	5,943	87.5	0.2
*Cutaneotrichosporon daszewskae* JCM 11166	BEDX00000000	PRJDB5901	Skin/Germany	Illumina MiSeq	17,225,847	12	2,186,752	60.98	8,162	199	7,963	1,697	6,266	85.2	0.5
*Cutaneotrichosporon dermatis* JCM 11170	BCKR00000000	PRJDB3725	Human infected skin/Tübingen, Germany	HiSeq. 2500	23,337,637	37	2,690,106	60.05	8,945	297	8,648	511	8,137	92.1	0.3
*Cutaneotrichosporon mucoides* JCM 9939^T^	BCJT00000000	PRJDB3710	Meningitis case/Belgium	HiSeq. 2500	40,783,511	84	1,668,497	60.14	15,434	507	14,927	1,016	13,911	94.4	**60.8**
*Cutaneotrichosporon oleaginosum* ATCC 20509^T^	MATS00000000	PRJNA327102	Dairy plant	PacBio	19,908,169	16	2,509,747	60.59	8,302	266	8,036	169	7,867	89.6	0.4
*Cutaneotrichosporon oleaginosum* IBC0246	JZUH00000000	PRJNA239490	-	Illumina	19,835,558	180	216,041	60.74	8,267	267	8,000	197	7,803	89.1	0.5
*Trichosporon asahii* JCM 2466^T^	BCLT00000000	PRJDB3696	Human, Japan	HiSeq. 2500	24,687,929	36	2,256,092	59.45	9,439	502	8,937	210	8,727	94.4	1.1
*Trichosporon asahii var. asahii* CBS 2479^T^	ALBS00000000	PRJNA164647	Trichosporia cutis proriasiformis progressiva, Japan	454; Illumina	24,540,311	78	1,660,894	59.01	9,364	497	8,867	287	8,580	93.2	0.5
*Trichosporon asahii var. asahii* CBS 8904	AMBO00000000	PRJNA172216	Maize cobs^§^ Illinois Peoria USA	454; Illumina	25,299,608	194	3,223,897	58.91	9,816	518	9,298	810	8,488	93.7	0.7
*Trichosporon coremiiforme* JCM 2938^T^	JXYL00000000	PRJDB3697	Head lesion caused by beesting/Turrialba, Costa Rica	Illumina HiSeq	42,353,277	190	1,468,092	59.60	15,969	908	15,061	2,104	12,957	95.3	**69.5**
*Trichosporon faecale* JCM 2941^T^	JXYK00000000	PRJDB3698	Human feces	Illumina HiSeq	24,653,913	32	3,676,950	60.21	9,494	471	9,023	1,233	7,790	93.6	0.7
*Trichosporon inkin* JCM 9195	JXYM00000000	PRJDB3701	Human	Illumina HiSeq	20,339,538	18	2,739,924	62.69	7,700	318	7,382	654	6,728	89.3	0.2
*Trichosporon ovoides* JCM 9940	JXYN00000000	PRJDB3702	White piedra infected scalp	Illumina HiSeq	40,322,879	116	2,824,449	60.22	15,163	584	14,579	2,470	12,109	96.4	**70**
*Pascua guehoae* JCM 10690	BCJX00000000	PRJDB3714	Meadow soil/Renswoude, the Netherlands	HiSeq. 2500	33,698,914	35	2,162,681	59.17	11,097	1,032	10,065	3,277	6,788	91.9	1.0
*Prillingera fragicola* JCM 1530^T^	BEDY00000000	PRJDB5902	Strawberry /Market in Akihabara, Tokyo, Japan	Illumina MiSeq	20,263,689	21	1,928,959	58.21	8,282	297	7,985	1,620	6,365	94	0.7
*Vanrija humicola* JCM 1457^T^	BCJF00000000	PRJDB3692	Soil	HiSeq. 2500	22,653,840	10	3,082,120	62.74	9,255	279	8,976	495	8,481	93.7	0.4
*Vanrija humicola* UJ1	BFAH00000000	PRJDB6593	Soil	Illumina HiSeq. 2500	22,628,423	46	1,340,400	62.76	9,270	296	8,974	483	8,491	93.5	0.4
*Takashimella koratensis* JCM 12878^T^	BCKT00000000	PRJDB3728	Leaf of Lagerstroemia calyculata/Thailand	HiSeq. 2500	25,142,362	31	1,454,648	54.94	9,382	54	9,328	3,020	6,308	95.1	0.2
*Takashimella tepidaria* JCM 11965^T^	BCKS00000000	PRJDB3726	Stream water/Japan	HiSeq. 2500	22,370,450	44	1,186,041	44.66	6,989	59	6,930	1,035	5,895	90	0.2

*Completeness and ^¥^duplication determined using BUSCO based on basidiomycota_odb9. ^§^Human pathogenic (superficial and systemic).

While the *Trichosporonaceae* include several opportunistic human pathogens, there has also been increased interest in these taxa for a broad range of biotechnological applications. Most pertinently, members of the *Trichosporonaceae* are known to produce and accumulate large amounts of single cell oil (SCO) relative to their dry biomass^5–11^, with up to 70% w/dw_biomass_ (weight/dry weight of biomass) accumulated by *Cutaneotrichosporon oleaginosus*^12^. Furthermore, they are amenable to large-scale fermentations as they are not as sensitive as other oleaginous yeasts to fermentation inhibitors including furanes and phenolic compounds^8^. These factors make members of the Trichosporonales suitable candidates in a wide range of biotechnological applications such as the production of oleo-chemicals and biofuels^13,14^.

The rapid development of genome sequencing technologies and bioinformatics has been pivotal in shaping our understanding of fungal genetics. Since the publication of the first fungal genome, *Saccharomyces cerevisiae*, in 1996^15^, fungal genomics has experienced rapid development. As of June 2019, 5,269 fungal genome assemblies have been deposited in the NCBI database^16^. With the increasing availability of fungal genomes, recent works have harnessed the information contained in the genomes to develop more robust taxonomic frameworks for several fungal taxa. For instance, Takashima *et al*.^17–19^ have pioneered and variously reported a genome-based characterisation and phylogenetic analysis of the order Trichosporonales using 24 haploid and 3 natural hybrid genomes. Furthermore genome sequencing provides access to the full complement of proteins encoded on a fungal genome, which can serve as resource for modelling functional capacities of the fungal strains and to further their use as biological resources in a wide range of biotechnological applications^20^.

In the current study, we have employed comparative genomic strategies to study thirty-three members of the family *Trichosporonaceae*. Phylogenomic analysis identified three mis-classified taxa within this family, while genes coding for enzymes involved in oleagenicity and their regulatory regions show evolutionary patterns distinct from the genome scale phylogeny. Furthermore, the genome comparisons highlighted a range of genetic determinants underlying the distinct lifestyles and niche specialisations of the different taxa within this family.

## Results and Discussion

### Genomic characteristics of the *Trichosporonaceae*

The genomes of thirty-three taxa belonging to the genera *Apiotrichum* (nine strains), *Cutaneotrichosporon* (twelve strains), *Pascua guehoae*, *Prillingera fragicola*, *Trichosporon* (eight strains) and *Vanrija* (two strains) were incorporated in the analyses. Twenty-nine of the strains have haploid genome structures, while three strains, namely *C. mucoides* JCM 9939^T^, *T. ovoides* JCM 9940 and *T. coremiiforme* JCM 2938^T^, have been shown to comprise hybrid genomes^18^. In this study, genome duplication and phylogenetic analyses revealed one additional strain, *C. cutaneum* B3 to comprise of a hybrid genome. Two strains of *Takashimella* (belonging to the closely related family *Tetragoniomycetaceae* were included as outgroups. A survey of the origin of the *Trichosporonaceae* strains shows a wide geographic distribution of the organisms with isolates obtained from food, decomposing wood, human body, soils, water bodies, among others (Table 1). The two outgroup strains have originated from two distinct sources; leaf of plant and stream water. However, majority of members of the genus *Trichosporon* and *Cutaneotrichosporon* species, for which the genome sequences are available, are either associated with human or animal skin while genomes of isolates from insect^1,21^ are not available. This may reflect preference for the sequencing of clinically important strains. The phylogenomic analyses of thirty-three members of the family *Trichosporonaceae*, including *Apiotrichum porosum* DSM 27194 and one putative hybrid genome strain, *C. cutaneum* B3 are presented here. The estimated genome sizes of the thirty-three *Trichosporonaceae* strains ranged between 16.4 and 42.4 Mb with an average G + C content range of 56.5–62.8%. The N50, which is the contig/scaffold size for which at least 50% of the assembly is contained in equal or larger contigs/scaffolds, ranged between 53.5Kb in *T. akiyoshidainum* HP2023 and 5.6 Mb in *C. cutaneum* ACCC 20271, indicating wide variety in assembly quality. However, previous studies have shown that large N50 values may arise because of erroneous concatenation of contigs, thereby limiting the value of this metric in evaluating assembly quality^22^. The largest genome sizes (average of 40.5 Mb) are observed for the four hybrid genomes incorporated in the analysis. Among the haploid genomes, the largest genome sizes belong to the yeast strains that are predominantly isolated from various soil types. Prediction of protein encoding gene models revealed that the genomes of these fungi encode between 6,477 (*C. curvatus* SBUG-Y 855) and 15,061 (*T. coremiiforme* JCM 2938^T^) proteins. Evaluation of the predicted protein models using the BUSCO^23^ basidiomycota_odb9, which includes 1335 single copy genes/proteins, revealed that the genome completeness of the yeast strains included in this study ranged between 80.8 and 97.2% (Table 1). Additionally, BUSCO^23^ analysis revealed extensive protein duplication ranging between 55.7 to 70% in the four hybrid genomes that harbour the largest genome sizes. In contrast, the two outgroup species have genome sizes of 22.4 and 25.1 Mb and G + C content of 44.66 and 54.94% for *Takashimella tepidaria* JCM 11965^T^ and *T. koratensis* JCM 12878^T^, respectively.

### Genome-wide phylogenetic analysis reveals several misclassifications in the *Trichosporonaceae*

**Figure 1:**
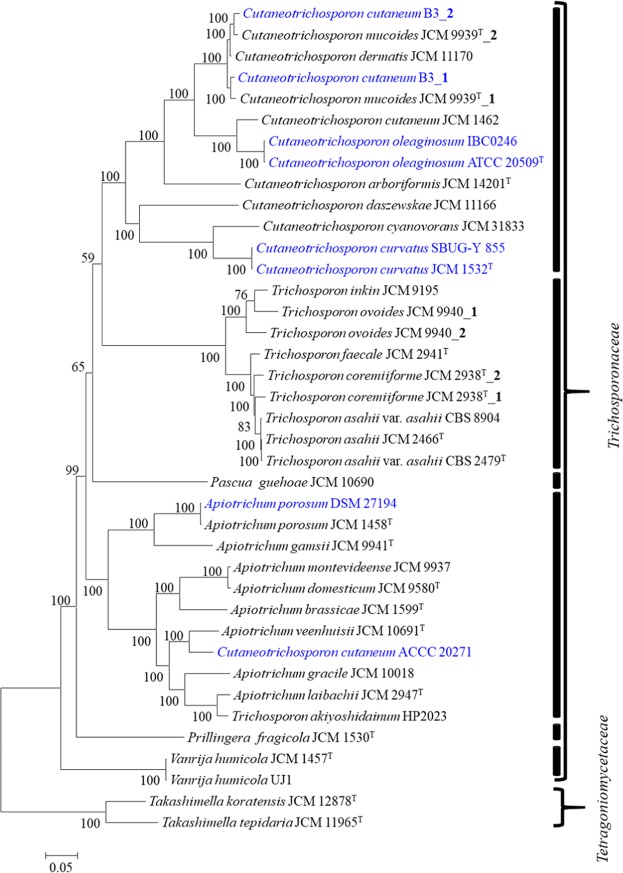
Phylogenomic analysis of members of the family *Trichosporonaceae*. The maximum likelihood (ML) tree was inferred from the concatenated protein alignment (223,082 amino acids) of 405 proteins present in single copies among the haploid genomes and only in duplicate copies in the hybrid genomes. The phylogeny was generated using IQ-TREE version 1.6.7 based on the LG + F + R10 model. The ML was generated with confidence values based on 1,000 bootstrap replicates. The documented oil accumulating members of the family are indicated in blue fonts. The labels ‘_1’ and ‘_2’ indicate the two sets of single copy orthologs (SCOs) in the hybrid genomes, where the letter shows higher amino acid similarity to the closest haploid genome.

Orthologous proteins conserved among all compared taxa were identified using Proteinortho5^24^. A total of 1,351 proteins are common to all the studied strains, including the outgroups. However, to put the hybrid genomes into phylogenomic perspective, 405 orthologous proteins present solely in single copies among the haploid genomes and only in duplicate copies in the hybrid genomes were used to reconstruct the phylogeny of the *Trichosporonaceae*. The trimmed concatenated protein alignment comprised 223,082 amino acids in length. The resultant maximum likelihood phylogeny (Fig. 1) shows the clustering of the *Trichosporonaceae* into six distinct clades. Eleven of the twelve *Cuteaneosporotrichon*, seven of the eight *Trichosporon* and all nine *Apiotrichum* strains incorporated in the study fall into three separate clades congruent with the distinct *Trichosporonaceae* genera that they represent^1,2^.

While three clear genus clades can be observed in the single copy orthologues phylogeny (SCOP), two taxa, namely *C. cutaneum* ACCC 20271 and *T. akiyoshidainum* HP2023 are clearly delineated within the *Apiotrichum* clade in the SCOP, and should thus be reassigned to the latter genus. As has previously been observed through separation of subgenomes^18,25^, the duplicate orthologue copies (here referred to as ‘strain number’_1 and _2) in the three described hybrid genomes form distinct branches but are still retained within their genus clades (Fig. 1). When considering the fourth putative hybrid genome identified in this study, *C. cutaneum* B3, B3_1 clusters with *C. mucoides* JCM 9939^T^_1, while B3_2 also clusters with *C. mucoides* JCM 9939^T^_2, suggesting that the two strains are likely to have shared similar evolutionary history including episodes of hybridization. In addition to evidence from gene duplication (55.7%) determined using BUSCO^23^ basidiomycota_odb9, BLASTP analyses showed that *C. dermatis* JCM 11170 shares 92.41% and 97.92% amino acid similarity among the 405 single copy orthologues (SCO) with those of *C. cutaneum* B3_1 and B3_2, respectively. In additon, the 405 SCO sets of B3_1 and B3_2 shared on average 92.76% amino acid similarity, further proving support for the distinct origin of the duplicated single copy orthologue sets.

### Differences in the proteolytic and carbohydrate metabolic enzyme complements of the *Trichosporonaceae* may influence their lifestyles

**Figure 2:**
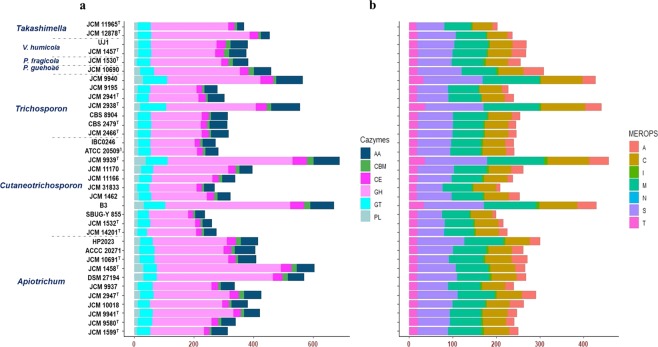
Comparison of number of proteins associated with (**a**) CAZymes and (**b**) MEROPS among thirty-three strains of *Trichosporonaceae*. CAZymes; AA: auxillary activities, CBM: carbohydrate-binding modules, CE: carbohydrate esterases, GH: glycoside hydrolases and GT: glycosyltransferases. MEROPS; A: aspartic peptidases, C: cysteine peptidases, M: metallo-peptidases, N: asparagine peptide lyases, S: serine peptidases, T: threonine peptidases, and I: protease inhibitors.

To further enhance our understanding of various functional and adaptational capacities of the studied strains, proteins annotated as Carbohydrate-Active enZYmes (CAZYmes) and proteolytic enzymes (MEROPS) were identified and compared (Fig. 2a). The presence of these proteins can provide an indication of the ranges of possible carbohydrate and protein substrates utilised by an organism. CAZYmes represent a broad scope of proteins associated with the assembly, modification and degradation of various types of carbohydrates^26^ and are curated in the Carbohydrate-Active EnZYmes database (http://www.cazy.org). The *Cutaneotrichosporon* strains displaying hybrid genomes showed the highest numbers of CAZYmes; 671 in *Cutaneotrichosporon cutaneum* B3 and 689 in *Cutaneotrichosporon mucoides* JCM 9939^T8^ (Supplementary Fig. 1) Aside from these hybrid genome taxa, the genomes of the two *Apiotrichum porosum* strains encode the highest numbers of CAZYmes (570 & 604 proteins) with ~68%, of these belonging to the class of glycoside hydrolases (GH). Similarly, GHs form the largest proportion of the CAZYmes in all studied strains. Considering the average CAZYme numbers within each genus, the *Apiotrichum* species also harbour the most CAZYmes (average 421), followed by *Vanrija* (379), *Trichosporon* (378) and *Cutaneotrichosporon* (365). However, the single available genome of *Pascua guehoae* also encodes 460 CAZYmes. Within the genera, *Trichosporon* has the highest average number of CAZYmes linked to auxillary activities (AA) and glycosyltransferases (GTs) encompassing 65 and 55, respectively and *Vanrija* harbours the highest average number of carbohydrate-binding modules (CBM) and carbohydrate esterases (CE) with 17 and 30, respectively while the highest mean number of glycoside hydrolases, 261 and polysaccharide lyases (PL), 19 was recorded in *Apiotrichum* and *Cutaneotrichosporon*, respectively. Abundance of CAZYmes has been linked to the various fungal adaptations with saprophytic fungi harbouring larger numbers of these enzymes compared to their parasitic counterparts^27^. This feature may readily be inferred from the current comparison, where on the average the free-living fungi of the genera *Apiotrichum* and *Vanrija* harbour greater numbers of CAZYmes than the predominantly host-associated *Trichosporon* and *Cutaneotrichosporon* taxa. Furthermore, the abundance of GHs and CEs^28^ in *Apiotrichum* and *Vanrija*, respectively may reflect their capacity to breakdown and utilise wide range of substrates. These taxa are frequently isolated from soil and other environments where they degrade and subsist on various forms of complex substrates^29^.

Proteolytic enzymes are proteins that hydrolyse peptide bonds and are widely distributed across all domains of life with estimates showing that they comprise ~2% of all proteins encoded on the genomes of organisms across all domains of life^30^. These enzymes form an important component of the biomass degradation capacities of both fungi and bacteria^31^ and their distribution is reflective of the lifestyle of the organisms. For instance, comparison of pathogenic and non-pathogenic *Pseudogymnoascus* strains revealed a marked underrepresentation of proteases in the former relative to the latter organisms^32^. To predict these enzymes, proteins of the organisms included in this study were searched against the manually curated enzymes in the MEROPS database^33^. All seven classes of MEROPS, namely aspartic peptidases (A), cysteine peptidases (C), metallo-peptidases (M), asparagine peptide lyases (N), serine peptidases (S), threonine peptidases (T), and protease inhibitors (I) are represented in the genomes of the thirty-three *Trichosporonaceae*, comprising approximately 3% of the proteins of the organisms (Fig. 2b). As observed with the CAZYmes, the hybrid genomes in the genera *Trichosporon* and *Cutaneotrichosporon* harbour the most abundant peptidases, ranging between 428 and 458 proteins. Omitting the hybrid genomes, the highest average number of the MEROPS was observed among the *Vanrija* and *Apiotrichum* species, with 264 and 270 proteins, respectively. The three most abundant MEROPS belong to the class S (56–143 proteins), M (63–130 proteins) and C (49–101 proteins) across the different genera. However, asparagine peptide lyase (N), which is the only member of the MEROPS that is not a peptidase^34^, appears to be restricted to only five of the strains; *Apiotrichum domesticum* JCM 9580^T^ (1 protein), *Apiotrichum laibachii* JCM 2947^T^ (1 protein), *Cutaneotrichosporon arboriformis* JCM 14201^T^ (2 proteins), *Trichosporon faecale* JCM 2941^T^ (1 protein) and *Trichosporon inkin* JCM 9195 (1 protein). Serine and metallo-peptidases are widely distributed in fungi and may reflect the capacity of these organisms to use proteinaceous substrates^35,36^. However, serine peptidases contents have been shown to be determined by both proteome size and lifestyle of fungi. Parasitic fungi, often associated with reduced genomes/proteomes and those involved in symbiosis have been shown to harbour less serine proteases^37^. The predominance of serine peptidases S (average 81 and 82 proteins, respectively) and metallo-peptidases (average of 77 and 78 proteins, respectively) among the mainly soil inhabiting *Vanrija* and *Apiotrichum* spp. reflect their versatility in sequestering a wide range of complex substrates in their environment. Cysteine peptidase were reported as pivotal in sustaining parasitic lifestyles^38^. Among the *Trichosporonaceae*, the upper range of the cysteine peptidases are seen among the predominantly host-associated *Trichosporon* (an average 66 proteins) and *Cutaneotrichosporon* (on average 60 proteins) strains, while *Apiotrichum* spp. and *Vanrija* strains only had on average 56 and 53 of these proteins encoded on their genomes, respectively.

### Phylogeny of oleagenic proteins and promoter regions of their genes highlights the complex evolution of lipid biosynthetic pathway

**Figure 3:**
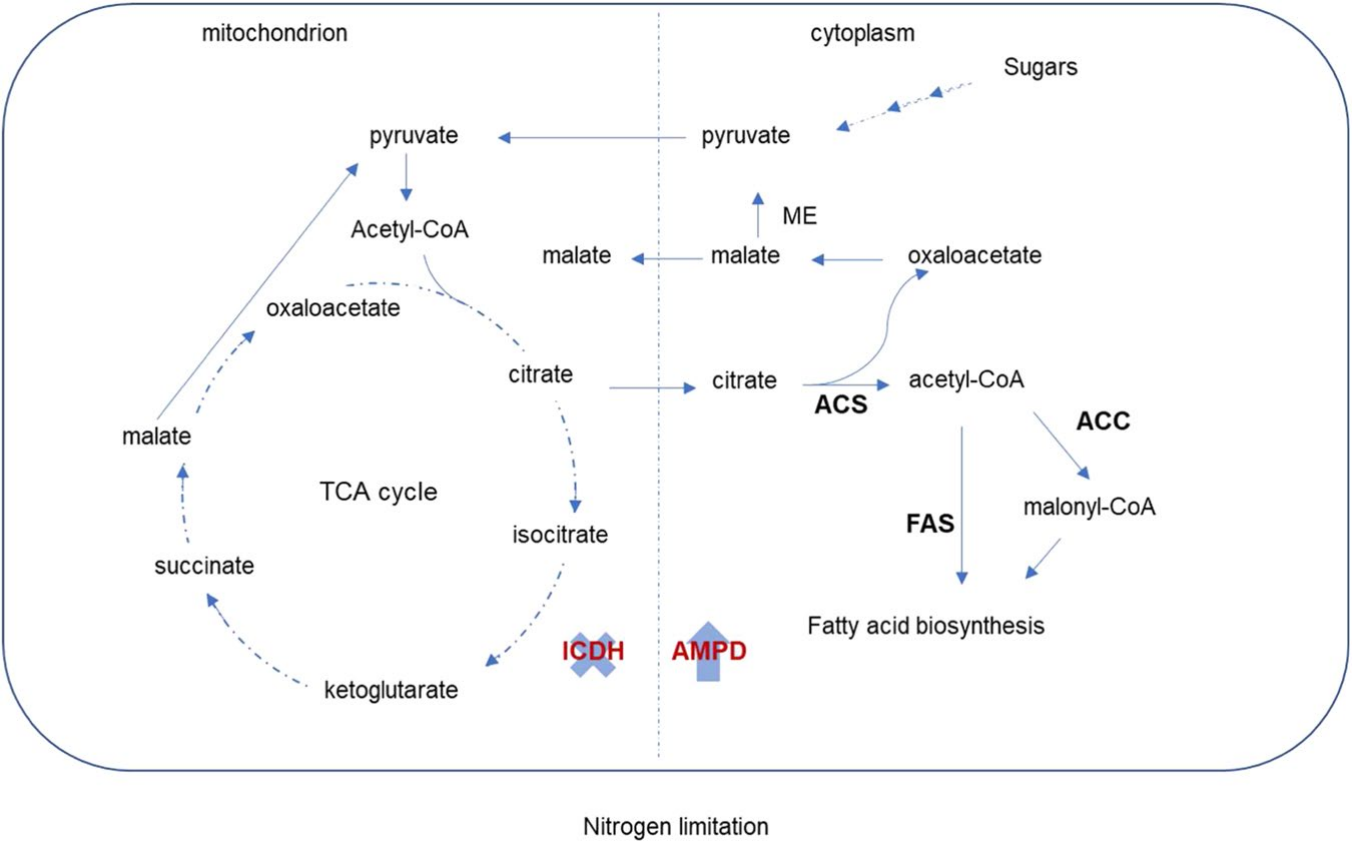
Illustration of the initiation of the biochemical oil production in yeasts showing the steps within the pathway catalysed by the studied enzymes under nitrogen limitation. ACC, acetyl-CoA carboxylase, AMPD, AMP deaminase, ACS, ATP-citrate synthase, FASI & II, fatty acid synthase subunits alpha and beta and ICDH, isocitrate dehydrogenase [NADP]. × and ↑ indicates the inhibition of ICDH and increased activity of AMPD under nitrogen limitation. Modified from^7^

The biochemical production and accumulation of single cell oil in fungi has received extensive interest because these organisms could serve as eco-friendly sources of lipids and other important biochemicals with a wide range of biotechnological applications^7,39^. To provide additional insights into the genomic basis of oil accumulation among the compared strains, six proteins involved in the biochemical pathway (Fig. 3) central to lipid production and accumulation were analysed. These were acetyl-CoA carboxylase (ACC), AMP deaminase (AMPD), ATP-citrate synthase (ACS), fatty acid synthase subunits alpha and beta (FASI & II) and isocitrate dehydrogenase [NADP] (ICDH). Understanding the structure of the regulatory elements of the genes that code for these proteins may be pivotal in deciphering approaches for enhanced oil production. For instance, an increase in lipid accumulation was achieved through the overexpression of ACC under various promoter systems^40,41^. As such, the transcription factor binding domains (TFBDs) 600 bp upstream of these genes were analysed.

Evaluation of the proteomes of the yeasts included in this study reveals that orthologues of the selected proteins occur in all of the strains studied, with the exception of *T. asahii* var. *asahii* CBS 8904 and *T. akiyoshidainum* HP2023 in which orthologues of ACC are absent and *C. curvatus* SBUG-Y 855, which does encode an orthologue of AMP on its genome. The hybrid genomes of *C. cutaneum* B3, *T. coremiiforme* JCM 2938*, T. ovoides* JCM 9940 and *C. mucoides* JCM 9939 harbour two copies of FASI &II, ACC and ACS. However, only JCM 2938 retains the duplicate copy of ICDH, while AMPD is present in two copies in B3 and JCM 2938. Given the essential nature of these proteins, it is likely that the absence of some of the orthologues is associated with the level of genome completeness rather than the lack of the affected function.

**Figure 4:**
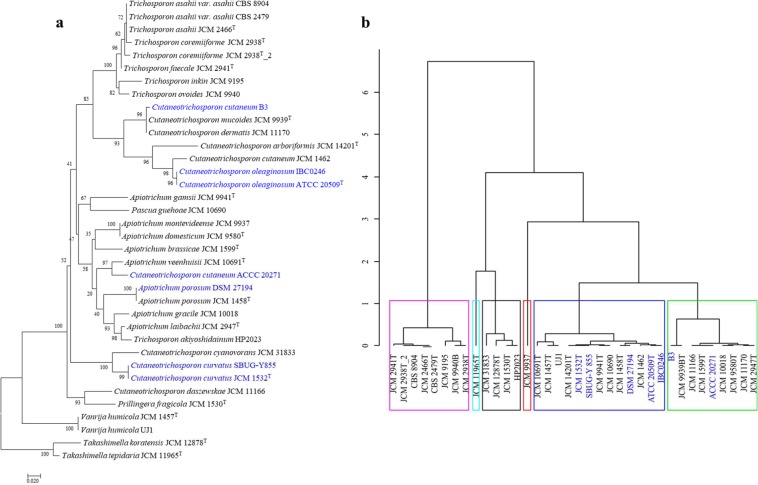
Evolutionary analyses of the ICDH protein and the upstream region of its gene among thirty-three strains of *Trichosporonaceae*. (**a**) ML tree of ICDH (380 amino acids long trimmed alignment) generated using IQ-TREE version 1.6.7 with confidence values based on 1,000 bootstrap replicates. (**b**) Distribution of predicted transcription factor binding sites 600 nucleotide bases upstream of the transcription initiation site of ICDH gene clustered using hierarchical clustering on principal components (HCPC) in R. The documented oil accumulating members of the family are indicated in blue fonts in the phylogeny and with blue arrows in the HCPC.

Oil production in yeasts has been linked to nutrient limitation, where the organisms channel carbon flux to lipid instead of energy production^5,7^. Two enzymes directly associated with this function are AMPD and ICDH with the former shown to enhance the depletion of AMP and consequently playing a role in the inhibition of ICDH^42^. Comparison of a phylogeny on the basis on the AMPD amino acid sequences (Supplementary Fig. 2,a) showed that, apart from the placement of *V. humicola*, this tree shows a similar topology and clustering as the SCOP. Clustering of the strains based on the distribution and abundance of TFBDs upstream of the AMPD gene (Supplementary Fig. 2,b) shows distinct grouping of the organisms suggesting disparate evolution of this regulatory region. In the ICDH tree (Fig. 4a), only the *Trichosporon* species showed a coherent grouping while members of the genus *Cutaneotrichosporon*, including the known oleaginous strain *C. curvatus* show incongruent branching pattern relative to the SCOP, indicating distinct evolutionary history of the ICDH gene. Comparison of the TFBDs of the ICDH gene revealed that these fungi form six distinct clusters (Fig. 4b) with the documented oleaginous strains *A. porosum*, *C. curvatus* and *C. oleaginosum*, clustering together thereby suggesting a possible similarity in the regulation of the ICDH gene among these strains. Two other reported oil accumulating yeast, namely *C. cutaneum* B3 and *C*. *cutaneum* ACCC 20271 are also closely clustered with the rest of the oleaginous strains. Discussion on the affiliation of the two strains has been presented above. The predicted TFBDs of ICDH include binding motifs for Gis1; Gat1p, Gln3p, Gzf3p; and Gln3p all of which have been implicated in the regulation of gene expression under nutrients starvation, including amino acids and nitrogen limitations^43–45^.

**Figure 5:**
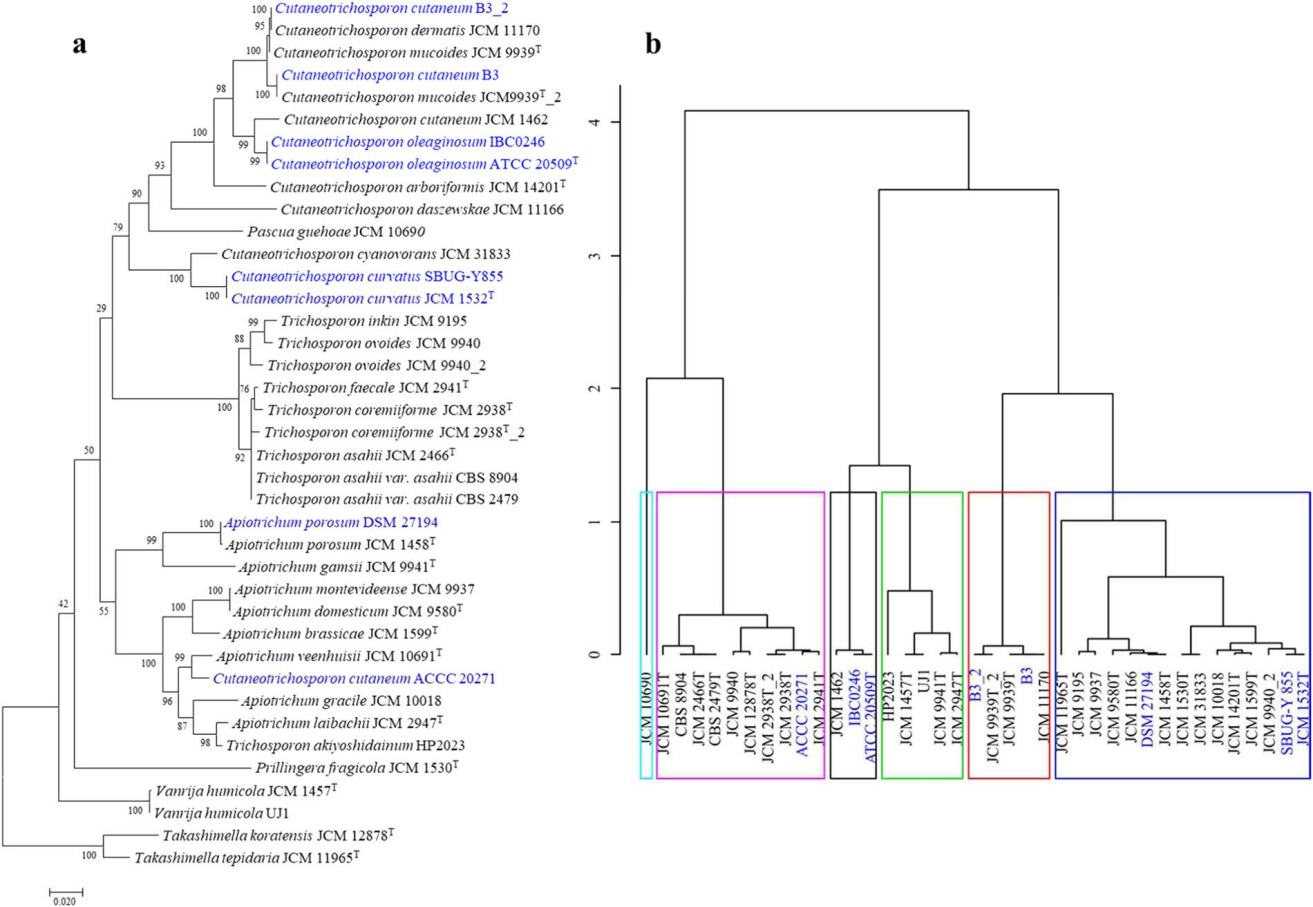
Evolutionary analyses of the ACS protein and the upstream region of its gene among thirty-three strains of *Trichosporonaceae*. (**a**) ML tree of ACS (1,097 amino acids long trimmed alignment) generated using IQ-TREE version 1.6.7 with confidence values based on 1,000 bootstrap replicates. (**b**) Distribution of predicted transcription factor binding sites 600 nucleotide bases upstream of the transcription initiation site of ACS gene clustered using hierarchical clustering on principal components (HCPC) in R. The documented oil accumulating members of the family are indicated in blue fonts in the phylogeny and with blue arrows in the HCPC.

Suppression of ICDH, which is considered as a feature specific to oleaginous yeasts^5^ results in the accumulation of citrate in the mitochondrion. The citrate is then transferred into the cytoplasm where ACS catalyses its conversion into to acetyl-CoA and oxaloacetate. Evaluation of the ACS phylogeny (Fig. 5a) showed similar branching pattern with the SCOP. However, *P. guehoae* is placed within the well supported *Cutaneotrichosporon* clade. However, based on the TFBDs of ACS, the strains group into six distinct clusters (Fig. 5b) with two of the known oleaginous strains, *A. porosum* and *C. curvatus*, clustering together. In addition to the Gis1p, Msn2p, Msn4p, Rph1p, YER130C binding domains, which are known to regulate gene expression under nutrients limitation and stress^45^, the regulatory region of ACS includes the Adr1p TFBD. Adr1p is a carbon source-responsive transcription factor involved in the regulation of genes associated with ethanol, glycerol, and fatty acid utilization and peroxisome biogenesis^46–48^. As reflected in the characteristic clustering of *A. porosum* and *C. curvatus*, each of the strains carries two putative binding sites for Adr1p compared to *C. oleaginosum* which harbours four such TFBDS.

**Figure 6:**
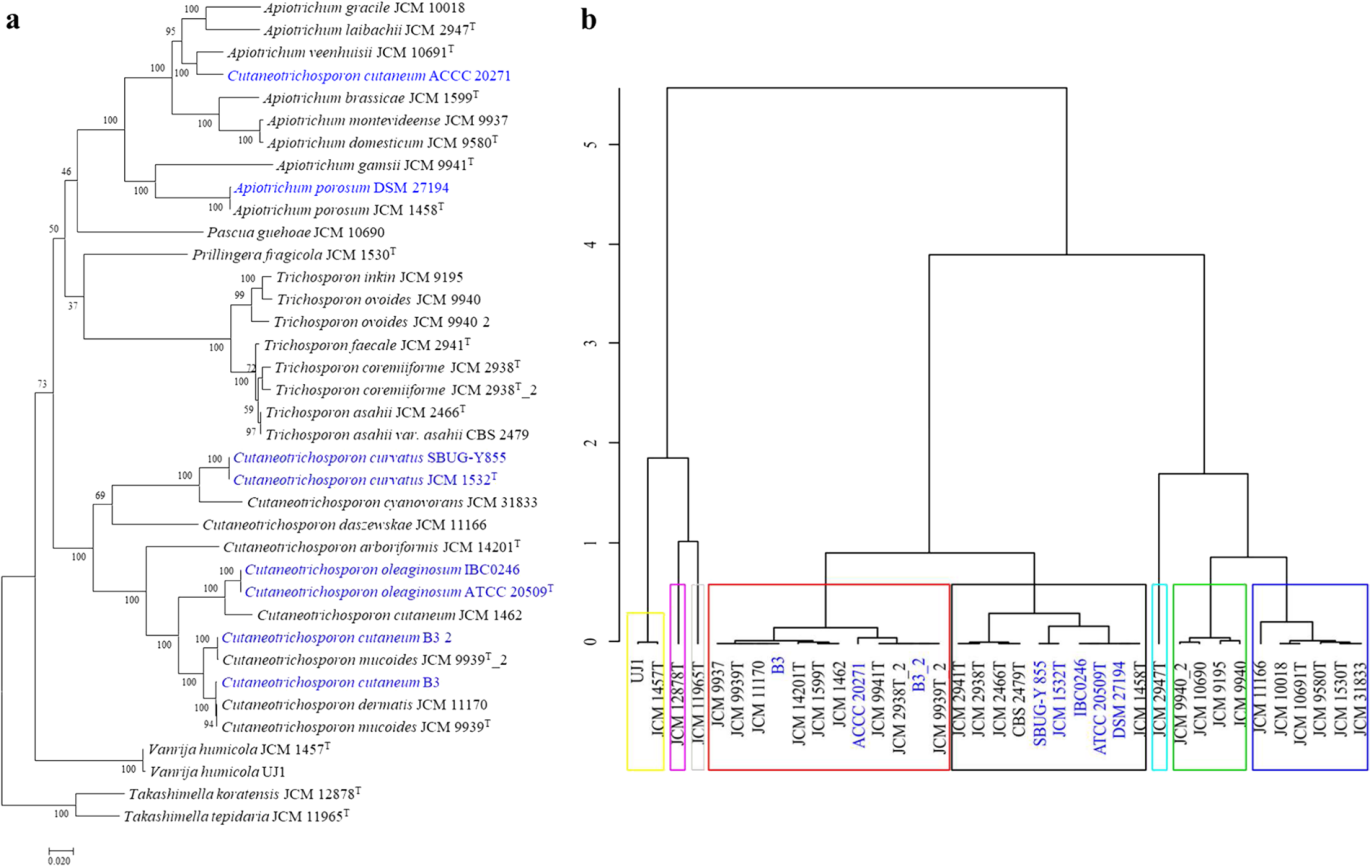
Evolutionary analyses of the ACC protein and the upstream region of its gene among thirty-three strains of *Trichosporonaceae*. (**a**) ML tree of ACC (2094 amino acids long trimmed alignment) generated using IQ-TREE version 1.6.7 with confidence values based on 1,000 bootstrap replicates. (**b**) Distribution of predicted transcription factor binding sites 600 nucleotide bases upstream of the transcription initiation site of ACC gene clustered using hierarchical clustering on principal components (HCPC) in R. The documented oil accumulating members of the family are indicated in blue fonts in the phylogeny and with blue arrows in the HCPC.

One of the products of the cleavage of citrate, acetyl-CoA, is either directly channelled to fatty acids synthesis via the FAS complex (catalysed by FASI &II) or converted into malonyl-CoA, which is subsequently directed to fatty acid synthesis. The latter reaction is catalysed by ACC. Incongruent with the SCOP, the ACC of *Apiotrichum* and *Trichosporon* species as well as those of *Pascua guehoae* and *Prillingera fragicola* appear to share similar evolutionary history clustering distinctly from the *Cutaneotrichosporon* species (Fig. 6a). The TFBDs of the ACC gene grouped the studied strains into eight distinct clusters (Fig. 6b). Based on this grouping, the five documented oleaginous yeasts assemble in two close clades. In addition to previously discussed putative sites for transcription factors regulating genes under nutrients limitation, adaptation to stress and utilisation of ethanol, glycerol, and fatty acid, the TFBDs of the ACC gene include a putative binding site for the zinc cluster protein Gsm1p and the basic helix-loop-helix transcription factor Pho4p. Gsm1p has been predicted to regulate energy metabolism^49,50^ while Pho4p was shown to be activated in response to phosphate limitation and controls genes of the phosphatase regulon and an inorganic phosphate (P_i_) transport system in *Saccharomyces cerevisiae*^51,52^. P_i_ limitation has been used as an alternative means of inducing oil accumulation in oleaginous yeast^53^. The phylogeny generated based on FAS subunits (Supplementary Fig. 3c,e) revealed a clustering similar to that observed in the SCOP with exception of the placements of *P. guehoae* and *P. fragicola* in both trees and the distinct grouping of *C. curvatus* and *C. cyanovorans* in FASII (Supplementary Fig. 2,f). This may indicate a disparate evolution of the FASII genes in the latter strains. In terms of the TFBDs, the oleaginous strains group in separate clusters for both FASI & II (Supplementary Fig. 2d,f), indicating a more complex evolution of these genomic regions. However, the TFBDs of both genes include Gis1p, Msn2p, Msn4p, Rph1p, YER130C binding sites which are involved in gene regulation under nutrient starvation^45^ while the FASI regulatory region harbours Adr1p^46–48^ and Gsm1p^49,50^ binding domains and that of FASII includes Pho4p^49,50^ TFBDs. On the overall, the prediction of the TFBDs could serve as a preliminary approach for the genomic exploration and identification of potential oleaginous yeast.

Clustering of the fungal isolates based on the regulatory regions of genes encoding the enzymes that determine oil production pathway may be useful in selecting strains with similar pattern of putative regulatory mechanisms for subsequent characterisation. Considering the TFBDs clustering pattern of ICDH and ACC, seven strains namely, *A. porosum* JCM 1458T, *A. gamsii* JCM 9941^T^, *A. brassicae* JCM 1599^T^, *A. laibachii* JCM 2947^T^, *C. arboriformis* JCM 14201^T^, *C. mucoides* JCM 9939^T^ and *C. dermatis* JCM 11170 are closely grouped with the oil accumulating isolates in the two clusters. Whereas the *Cutaneotrichosporon* species may not be excellent candidates because of their association with human host, the *Apiotrichum* species, all of which are free-living and isolated from various environments (Table 1) could potentially be oleagenic. *A*. *porosum* JCM 1458^T^ and *A. gamsii* JCM 9941^T^, are the closest relatives of the oleagenic *A*. *porosum* DSM 27194.

## Conclusion

Here, we have analysed the genomes of thirty-three members of the *Trichosporonaceae*, including five yeast, *A. porosum*, *C. curvatus*, *C. oleaginosum*, *C. cutaneum* B3 and *C*. *cutaneum* ACCC 20271 for which data regarding substantial lipid accumulation are available. Analysis of the whole genome phylogeny based on single copy orthologs shows that certain strains incorporated in the genera *Trichosporon* and *Cutaneotrichosporon* belong to the genus *Apiotrichum*. This highlights the need for the use of appropriate genomic evaluation schemes in the course of genome deposition in various databases. Comparison of the proteomes of these strains suggests functional diversification consistent with the various lifestyles and isolation sources of the studied organisms. For instance, abundance of the various CAZYmes and MEROPS signified the potential capacity of the yeast to degrade a wide variety of biomass, with distinct enzyme sets linked to these capacities in free-living and host-associated taxa within the *Trichosporonaceae*. The evaluation of selected genes coding for proteins involved in lipid biosynthesis and their corresponding transcription factor binding domains suggests a complex evolution with some level of conservation for the TFBDs of ACC, ACS and ICDH among the well-studied oil accumulating members of the family *Trichosporonaceae*. This indicates a possible similarity in terms of the regulation of the genes encoding these enzymes among the clustered strains. Further work should focus on investigating the specific binding potentials of the predicted TFBDs and their potential roles in oil production and accumulation in oleaginous yeast. Taken together, this information could be harnessed towards the selection of strains with potential functional capabilities that could be explored for the generation of environment friendly bioproducts, including single cell oils, biopharmaceuticals, and various raw materials in the food industry.

## Methods

### Genome sequences, gene predictions and annotation

Thirty-five genomes, comprising those of thirty-three members of the family *Trichosporonaceae* and two from the family *Tetragoniomycetaceae* (outgroup strains) were incorporated in this study (Table 1). Genome annotation was accomplished using the Funannotate pipeline (v. 1.5.0–8f86f8c)^54^. In brief, small duplicate contigs (*clean*) were removed, size sorted and renamed (*sort*) and repeat contains were masked using RepeatMasker v4.0.7 prior to gene prediction and annotation. Gene models were predicted using Augustus v3.2.3, GeneMark-ES v4.35, Evidence modeler v1.1.1 and tRNAscan-SE v1.3.1. For all gene prediction the Augustus training set for ‘cryptococcus’ was used. The predicted proteins were functionally annotated using Interproscan v.5.30–69.0, eggNOG-mapper v1.0.3.3-g3e22728, PFAM v.31.0, UniProtKB 2018_07, MEROPS v12.0, CAZYme (dbCAN v6.0), phobius v1.01 and SignalP v4.1. The completeness of the studied genomes was determined using BUSCO v3.0.3.

### Phylogenomic analysis

Single copy orthologues conserved among the predicted protein sequences of the thirty-three *Trichosporonaceae* and two outgroup strains were identified using Proteinortho5^24^ using all default parameters except percent amino acid identity which was set at 40%. To restrict the phylogeny to single copy orthologs (SCOs), the analysis included only proteins occurring in single copies among the haploid genomes and strictly in two copies for the hybrid genomes. The subgenome SCOs complement for each hybrid genome was determined by BLASTP comparison of the duplicate SCOs with the corresponding SCOs of the closest relative non-hybrid genomes^18,25^. The orthologous proteins were aligned using T-coffee v11.00.8cbe486^55,56^. The resultant alignment was concatenated and trimmed using Gblocks v0.9b^57,58^ with -b5 = h. The trimmed alignment was used to construct a Maximum likelihood (ML) tree using IQ-TREE version 1.6.7^59^ based on the LG + F + R10 model (predicted using IQ-TREE) and 1,000 bootstrap replicates.

### Evolutionary analysis of oleagenic proteins and promoter regions of their genes

Orthologs of selected proteins that play a major role in the biochemical pathways of lipid production in yeasts were selected among the *Trichosporonaceae* and *Tetragoniomycetaceae* based on BLASTP (percent identify cutoff value of 40%) using Proteinortho5^24^. Individual orthologous proteins were aligned using T-coffee v11.00.8cbe486^55,56^ and manually inspected to ensure accuracy of the alignments. The alignments were trimmed using Gblocks v0.9b^57,58^ and Maximum likelihood (ML) trees were generated using IQ-TREE version 1.6.7^59^ with 1,000 bootstrap replicates. Bedtools v2.27.1^60^ was employed to extract the regulatory regions of the genes encoding these proteins comprising 600 nucleotide bases upstream of the transcription initiation site. Each set of the regulatory regions was scanned for putative transcription factor binding domains (TFBDs) using the tools in YEASTRACT^61^, a database that curates the transcription factors (TF) and their target regulatory binding sites in *Saccharomyces cerevisiae*. The variation in the distribution of the TFBDs among the studied strains was used to group them using hierarchical clustering on principal components (HCPC) computed in R.

## Supplementary information

Supplementary data.
