# Supplementary material for: Genomic insights into the lifestyles, functional capacities and oleagenicity of members of the fungal family Trichosporonaceae
Source: Sci Rep. 2020 Feb 17;10:2780. doi: 10.1038/s41598-020-59672-2 (PMC7026411; doi:10.1038/s41598-020-59672-2)
Supplement: Supplementary file 1 — Supplementary data. [file 41598_2020_59672_MOESM1_ESM.docx]

**Genomic insights into the lifestyles, functional capacities and** **oleagenicity of members of the fungal family** ***Trichosporonaceae***

Habibu Aliyu^1^*, Olga Gorte^1^, Pieter de Maayer^2^, Anke Neumann^1^, Katrin Ochsenreither^1^*

^1^ Institute of Process engineering in Life Science 2: Technical Biology, Karlsruhe Institute of Technology, Germany

^2^ School of Molecular & Cell Biology, Faculty of Science, University of the Witwatersrand, WITS 2050 Johannesburg, South Africa

*Correspondence: habibu.aliyu@partner.kit.edu; [katrin.ochsenreither@kit.edu](mailto:katrin.ochsenreither@kit.edu)

**Supplementary Figures**


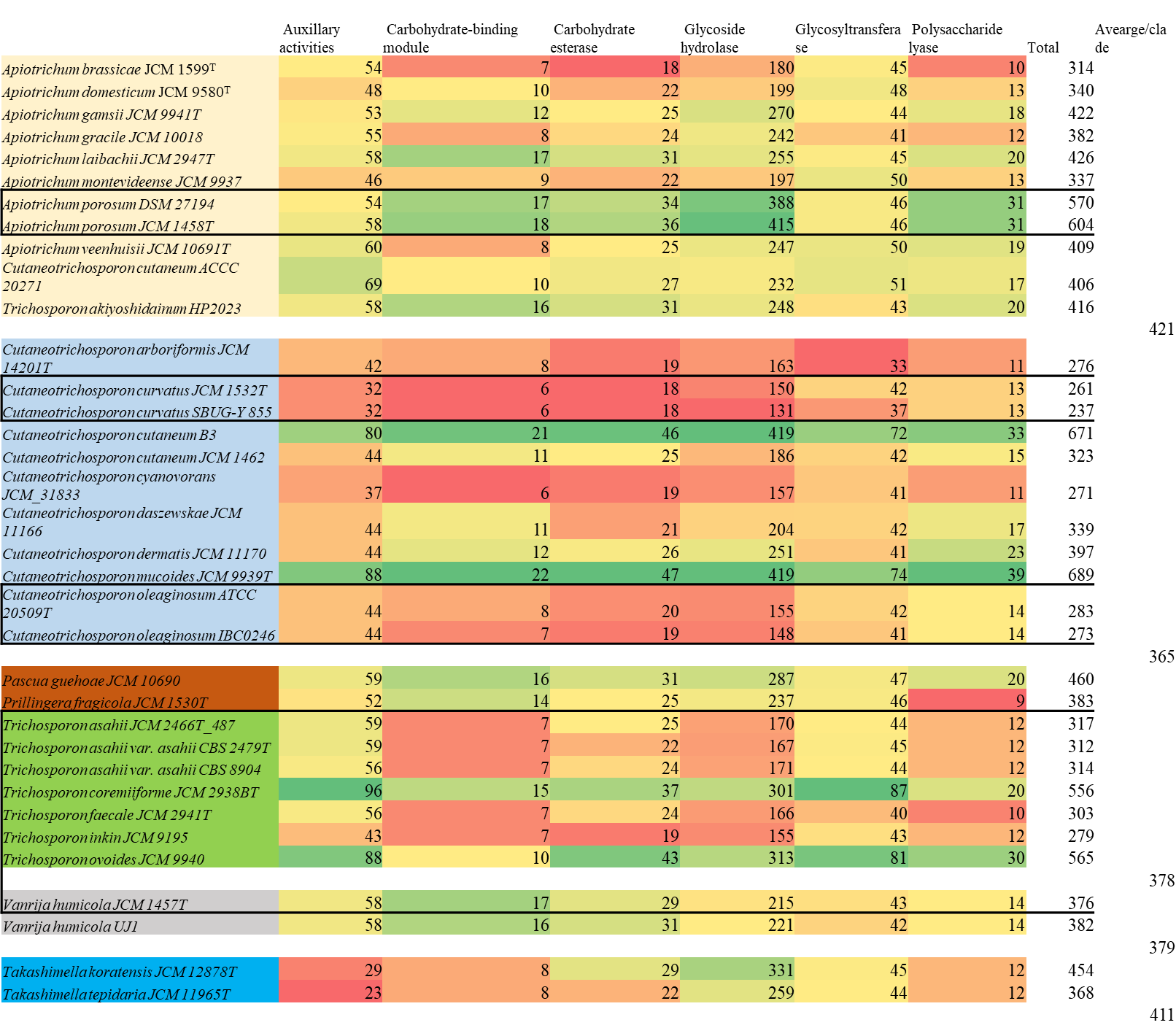


Supplementary Figure 1: Comparison of carbohydrate active enzymes (CAZYmes) among members of the family *Trichosporonaceae*


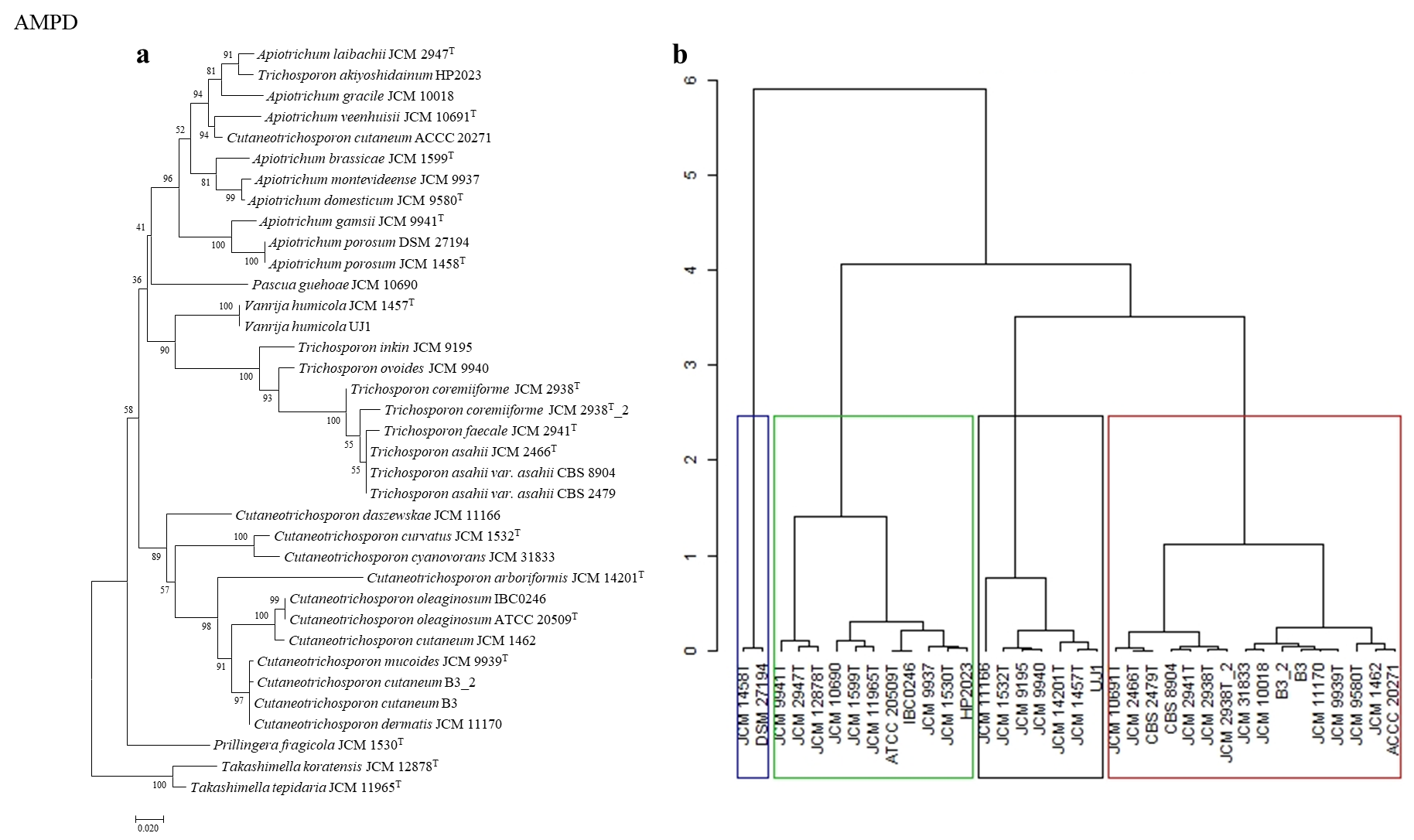


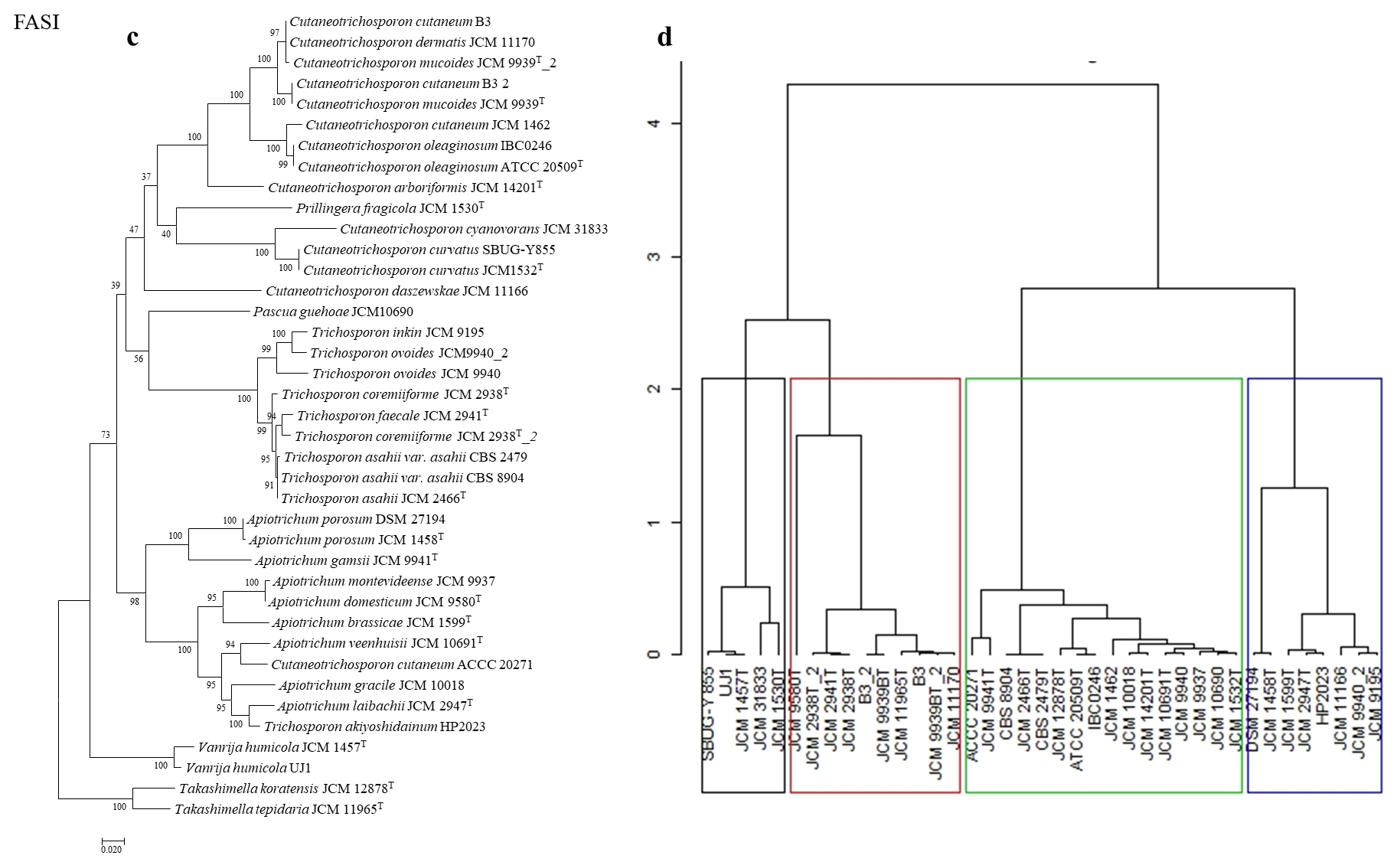


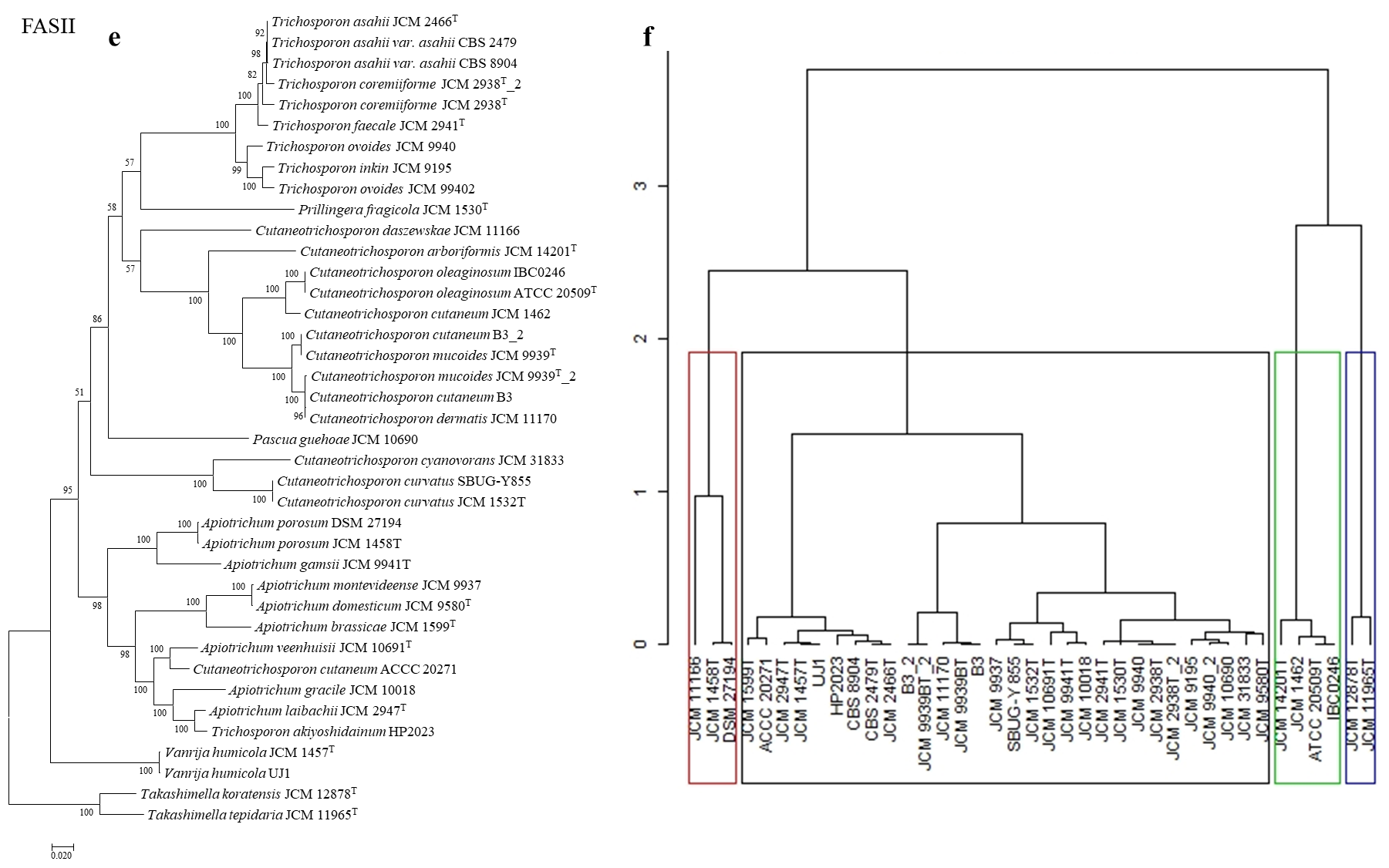


Supplementary Figure 2. Evolutionary analyses of the AMD, FASI & FASII proteins and the upstream region of their respective genes among thirty-three strains of *Trichosporonaceae*. (a, c & e) ML trees of AMPD (457 amino acids long trimmed alignment), FASI (994 amino acids long trimmed alignment) & FASII (2,305 amino acids long trimmed alignment) generated using IQ-TREE version 1.6.7 with confidence values based on 1,000 bootstrap replicates. (b, d & f). Distribution of predicted transcription factor binding sites 600 nucleotide bases upstream of the transcription initiation site of AMD, FASI & FASII genes clustered using hierarchical clustering on principal components (HCPC) in R.
